# Identification and characterization of multiple novel picornaviruses in fecal samples of bar-headed goose

**DOI:** 10.3389/fmicb.2024.1440801

**Published:** 2024-07-26

**Authors:** Yijie Sun, Yan Wang, Li Ji, Qifan Zhao, Quan Shen, Xiaochun Wang, Yuwei Liu, Likai Ji, Shixing Yang, Wen Zhang

**Affiliations:** School of Medicine, Jiangsu University, Zhenjiang, Jiangsu, China

**Keywords:** viral metagenomics, bar-headed goose, genomic structure, phylogenetic analysis, recombinant analysis

## Abstract

**Introduction:**

The bar-headed goose (*Anser indicus*), one of the most well-known high-altitude birds, is renowned for its adaptation to high-altitude environments. Previous studies have shown that they can be infected with highly pathogenic avian influenza; however, there is currently limited research on other viruses in bar-headed geese.

**Methods:**

In this study, 10 fecal samples of healthy bar-headed geese were collected, and viral metagenomics method was conducted to identify novel picornaviruses.

**Results:**

Seven novel picornaviruses were identified in the fecal samples of bar-headed geese. Most of these picornaviruses were genetically different from other currently known viruses in the NCBI dataset. Among them, PICV4 was determined to be a new species belonging to the *Anativirus* genus, PICV5 and PICV13 were classified as novel species belonging to the *Hepatovirus* genus, and the remaining four picornaviruses (PICV1, PICV19, PICV21, and PICV22) were identified as part of the Megrivirus A species of the *Megrivirus* genus. Recombinant analysis indicates that PICV21 was a potential recombinant, and the major and minor parents were PICV1 and PICV22, respectively.

**Conclusion:**

The findings of this study increase our understanding of the diversity of picornaviruses in bar-headed geese and provide practical viral genome information for the prevention and treatment of potential viral diseases affecting this species.

## Introduction

Birds can serve as both direct sources of infection for public health and animal populations, as well as vectors for a range of pathogens, encompassing avian-specific and zoonotic diseases. This dual role makes them pivotal in the transmission of microorganisms across different localities, influencing ecological dynamics and driving the evolution of numerous viral and bacterial strains (Chan et al., 2013). The bar-headed geese are a unique migratory bird species in Asia; they breed in some wetlands on the high plateaus of central Asia and winter in south-central Tibet (Scott et al., 2009; Meir et al., 2019). Due to the bar-headed goose being one of the main waterfowl species in the wetlands of the Qinghai-Tibet Plateau, people have taken various effective measures to protect it, such as location monitoring and habitat conservation (Wang et al., 2016). As migratory birds, the bar-headed geese may become infected with viruses from their habitats (such as water sources) or other animals in contact during migration. Previous studies have shown that bar-headed geese can be infected with the highly pathogenic avian influenza virus (Meng et al., 2019). However, currently, there is limited knowledge about the other viruses that bar-headed geese may carry and infect.

Members of the *Picornaviridae* family are small, positive-sense single-stranded RNA viruses with a genome length ranging from approximately 7.2 to 9.4 kb (Jiang et al., 2014). The *Picornaviridae* family comprises a total of 158 species belonging to 68 different genera. Picornaviruses are ubiquitous and distributed globally. Some picornavirus infections may induce diseases of the central nervous system, of the respiratory and gastrointestinal tracts, and of the heart, liver, pancreas, skin, and eye (Stanway et al., 2000; Tapparel et al., 2013; Vaughan et al., 2014; Sarry et al., 2022). Many picornaviruses had been detected in various types of samples from different birds that showed subclinical or clinical disease symptoms (Boros et al., 2017; Wille et al., 2021). At present, little is known about the diversity, host spectrum, pathogenicity, and factors that affect the co-circulation and co-infection of picornaviruses circulating in wild bar-headed geese.

With the rapid development of Next-Generation Sequencing (NGS) technology, the viral metagenomic approach has emerged as a powerful tool for investigating both novel and known viruses (Shan et al., 2022). The current study was designed to detect and characterize picornaviruses present in wild bar-headed geese. Multiple picornaviruses belonging to three different genera were identified in the fecal samples of bar-headed geese using viral metagenomics. The findings of this study increase our understanding of the diversity of picornaviruses in bar-headed geese and provide practical viral genome information for the prevention and treatment of potential viral diseases affecting this species.

## Materials and methods

### Sample collection and preparation

In 2020, with the assistance of animal experts, 10 healthy bar-headed geese were successfully captured using a cannon net in Saya County, Shigatse City, Tibet, China. Subsequently, fecal samples were collected using disposable, absorbent cotton swabs and shipped to our laboratory on dry ice. These samples were resuspended in 2 milliliters of Dulbecco’s Phosphate-Buffered Saline (DPBS) and vigorously vortexed for 10 min, followed by three freeze-thaw cycles. After centrifugation (15,000 × *g*, 4°C, 10 min), the supernatant of each sample was collected in a new 1.5 milliliters centrifuge tube and stored at −80°C for further use. Ethical approvals were given by the Ethics Committee of Jiangsu University with the reference number 2018ujs18023. Sample collection was performed in accordance with the Wildlife Protection Law of the People’s Republic of China. After sampling, the bar-headed geese were released without any damage.

### Viral nucleic acid extraction

A total of 500 μL of fecal suspension (50 μL of fecal supernatant from each fecal sample) was pooled together and filtered through a 0.45 μm filter (Merck Millipore, Billerica, MA, USA) to remove bacterial and eukaryotic cell-sized particles. The filtrates were then treated with a mixture of nuclease enzymes to digest the unprotected nucleic acids at 37°C for 90 min. Viral RNA and DNA were extracted by using the QIAamp MinElute Virus Spin Kit (Qiagen, Hilden, NRW, Germany) according to the manufacturer’s improved protocol. The concentrations of DNA and RNA were calculated using the Qubit 4 (Invitrogen, Carlsbad, CA, USA) nucleic acid concentration sequencer. The RNA and DNA were stored at −80°C for further use.

### Library construction and bioinformatics analysis

The viral nucleic acid pool containing DNA and RNA viral sequences was subjected to RT reactions with SuperScript III reverse transcriptase (Invitrogen, CA, USA) using 100 pmol of a random hexamer primer. The RT reaction conditions are 25°C for 10 min, 50°C for 60 min, 85°C for 5 min, and 95°C for 2 min. Then, the reaction products were quickly removed and placed on ice for > 2 min. The klenow enzyme (New England Biolabs, MA, USA) was used to generate the complementary chain of cDNA. The Klenow reaction conditions are 37°C for 60 min, 75°C for 20 min. Next, libraries were constructed using the Nextera XT DNA Sample Preparation Kit (Illumina, San Diego, CA, USA) according to the manufacturer’s protocol. A brief summary of the process includes adding connector primers and conducting 15 cycles of limited amplification. The library sequencing was completed by the Personalbio company using the NovaSeq Illumina platform with 250 base-paired ends with dual barcoding for each pool (Luo et al., 2012).

For bioinformatics analysis, the paired-end reads of 250 bp generated by NovaSeq were debarcoded using the vendor software from Illumina, which was processed using an in-house analysis pipeline running on a 32-node Linux cluster. Low-sequencing-quality tails were trimmed using a Phred quality score threshold of 10. Adaptors were trimmed using the default parameters of VecScreen, which is an NCBI BLASTn program with specialized parameters designed for adaptor removal. Bacterial reads were subtracted by mapping them to bacterial nucleotide sequences from the BLAST NT database using Bowtie2 v2.2.4. The cleaned reads were then de novo assembled by SOAPdenovo2 version r240 using a Kmer size of 63 with default settings. The assembled contigs, along with singlets, were aligned to an in-house viral proteome database using BLASTx (v.2.2.7) with an E-value cutoff of < 10^–5^. The candidate viral hits were compared to an in-house nonvirus nonredundant (NVNR) protein database to remove false-positive viral hits. The NVNR database was compiled using nonviral protein sequences extracted from the NCBI NR Fasta file and was based on a taxonomy annotation excluding the virus kingdom.

### Phylogenetic analysis

The analysis of evolutionary relationships was carried out using amino acid sequences predicted from the genomic data, with reference to the closest viral relatives determined by the best BLASTx hit and representative members of related viral species or genera. Sequence alignment was conducted with Clustal W in MEGA-X using the default settings (Kumar et al., 2018). Phylogenetic trees were constructed using MrBayes v3.2.7. The parameter “prset aamodelpr = mixed” was employed to enable the program to use the ten built-in amino acid models. The maximum number of generations was set to be ten million, and sampling occurred at every 50 generations, with the first 25% of Markov chain Monte Carlo (MCMC) samples being discarded during burn-in. Convergence was confirmed when the standard deviation of split frequencies was below 0.01. Bootstrap values were assigned to each node.

### Sequence alignment and ORF prediction

The pairwise comparison of viral amino acid sequences was conducted using SDTv1.2 software with default settings. Putative open reading frames (ORFs) in the genome were predicted using Geneious 11.1.2 software and the NCBI ORF finder.

### Recombination analysis

To test whether these megriviruses are recombinants, the complete genomes of all Megrivirus A strains available in the GenBank database were downloaded, then aligned using the Clustal W algorithm in the MEGA-X program together with these megriviruses. Using Recombination Detection Program v5.0 (RDP v5.0), the potential parental sequences and possible recombination sites were identified through different test methods, including RDP, Chimaera, MaxChi, SiSCan, GENECONV, BootScan, LARD, and 3Seq. Further recombinant analysis is performed using the BootScan program in the SimPlot software v3.5.1.

### Nucleotide sequence accession number

The complete viral genome sequences identified in this study were deposited in GenBank under the accession numbers PP827029 to PP827035. The raw sequence reads from the metagenomic library were deposited in the Sequence Read Archive of the GenBank database under accession number SRR29463689.

## Results

### Viral metagenomic overview

The library generated a total of 2,643,510 raw sequence reads on the Illumina NovaSeq platform. The average GC content (GC%) was 45.0%. Bioinformatics analysis revealed that 246,170 sequence reads had the best match with viral proteins. Among them, 118,682 sequence reads were matched to the family *Picornaviridae.*

### One novel species belonging to the genus *Anativirus* in the family *Picornaviridae*

**FIGURE 1:**
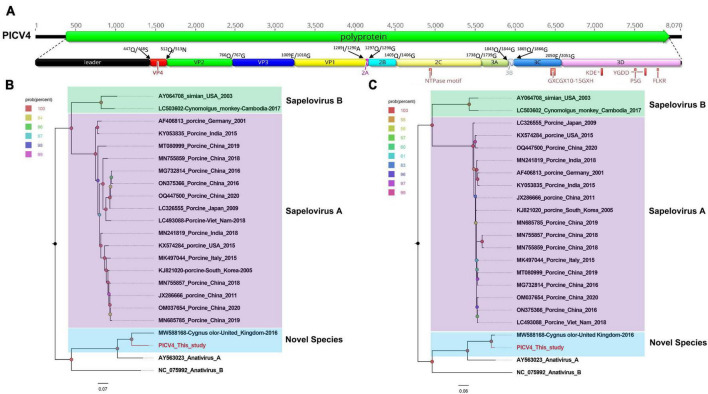
The genomic organization, conserved motifs, and phylogenetic analysis of the anativirus identified in bar-headed geese. **(A)** The genomic organization of one anativirus strain. The ORFs and viral encoding proteins of anativirus were marked with different colors. The conserved motifs were also shown. **(B,C)** The phylogenetic analysis based on the P1 region and 3CD of anativirus, which identified in this study, and other reference strains belonging to the *Anativirus* and *Sapelovirus* genus of the *Picornaviridae* family. PICV4 identified in this study was marked with red.

In this study, 7,532 sequence reads belonging to the genus *Anativirus* were identified. Using the “De Novo assemble” and “Map to Reference” programs in Geneious 11.1.2, one complete genome of anativirus was obtained and temporarily named PICV4 (assemble from contig 4) (mean coverage: 226.2). The genome of PICV4 is 8,070 nt (nucleotide) in length, which includes a 387-nt 5′ UTR (untranslated region), a 7,548-nt ORF (open reading frame), and an 135-nt 3′ UTR. The GC content of PICV4 is 42.0%. Like other anativiruses, the polyprotien of PICV4 can be divided into Leader, VP4, VP2, VP3, VP1, 2A–2C, and 3A–3D via a comparison with the polyprotein of the anativirus isolate TW90A (NC_006553) (Figure 1A). The P1 polypeptide of PICV4 consists of 1,289 aa (amino acid) and undergoes cleavage at the Leader/VP4 (Q^447^/S^448^), VP4/VP2 (Q^512^/N^513^), VP2/VP3 (Q^766^/G^767^), and VP3/VP1 (E^1,009^/G^1,010^). The leader protein is 447 aa in length and function unknown, while VP1-VP4 are viral structural proteins. The P2 polypeptide of 449 aa contains three nonstructural proteins, including 2A (cleavage site: Q^1297^/G^1298^), 2B (cleavage site: Q^1,405^/G^1,406^), and 2C. In the 2C protein of PICV4, there is a conserved NTPase motif (Figure 1A). The P3 polypeptide is 777 aa in length and cleaved into four nonstructural proteins, including 3A, 3B, 3C*^pro^* (protease), and 3D*^pol^* (RNA-dependent RNA polymerase) at sites 3A/3B (Q^1,843^/G^1,844^), 3B/3C (Q^1,865^/G^1,866^), and 3C/3D (E^2,050^/G^2,051^) (Supplementary Table 1). Several conserved proteinase and polymerase motifs, including GXCGX_10–15_GXH, KDE, YGDD, PSG, and FLKR, were presented in the 3C and 3D proteins of PICV4 (Figure 1A; Supplementary Table 2).

**FIGURE 2:**
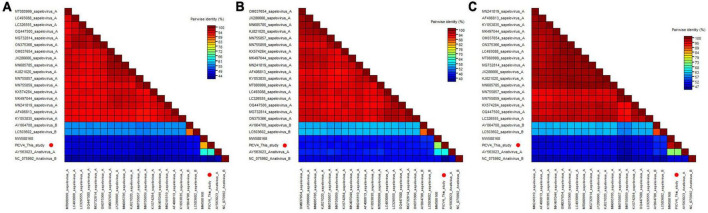
Pairwise comparison of amino acid sequences of one anativirus identified in this study with the representative strains of different species of the genus *Anativirus* and *Sapelovirus.* **(A)** Pairwise comparison based on polyproteins. **(B)** Pairwise comparison based on P1. **(C)** Pairwise comparison based on the regions of 2C+3CD. PICV4 identified in this study was marked with red solid circle.

To determine its genetic evolutionary relationship, two phylogenetic trees were constructed based on the P1 region and 3CD of PICV4, and the same regions of other representative sapeloviruses and anativiruses (Figures 1B, C). The results indicated that PICV4 clustered with one strain (MW588168) isolated from a fecal sample of *Cygnus olor* in the United Kingdom, forming a separate branch that was far away from the other two branches formed by members of Anativirus A and Anativirus B. Based on the results of phylogenetic trees, PICV4 may be a novel species of the *Anativirus* genus. To confirm this, amino acid sequence alignment was performed among the polyprotein, P1, and “2C+3CD” regions of PICV4 and other representative strains (Figure 2). The polyprotein alignment result showed that PICV4 shared the highest aa identity of 82.4% with the strain MW588168, while < 65.1% with other representative strains belonging to Sapelovirus A, Sapelovirus B, Anativirus A, and Anativirus B (Figure 2A). The P1 alignment result indicated that PICV4 has the highest aa identity of 69.6% with the strain MW588168 and < 60.7% with other representative strains (Figure 2B). The alignment result based on the “2C+3CD” region showed that PICV4 shared the highest aa identity of 96.1% with the strain MW588168 while < 72.4% with other representative strains (Figure 2C). Based on the ICTV classification standard, PICV4 should be classified as a novel species of the genus *Anativirus*.

### One novel species belonging to the genus *Hepatovirus* in the family *Picornaviridae*

**FIGURE 3:**
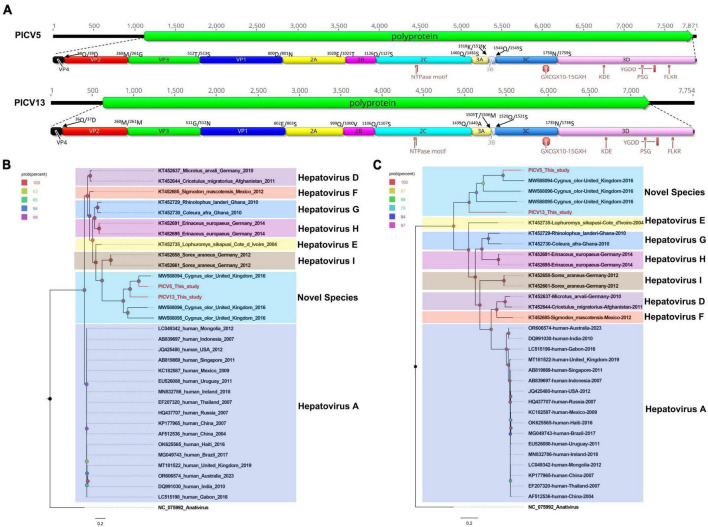
The genomic organization, conserved motifs, and phylogenetic analysis of these hepatoviruses identified in bar-headed geese. **(A)** The genomic organization of two hepatoviruses strains. The ORFs and viral encoding proteins of hepatoviruses were marked with different colors. The conserved motifs were also shown. **(B,C)** The phylogenetic analysis based on the P1 region and 3CD of hepatoviruses, which identified in this study, and other reference strains belonging to the *Hepatovirus* genus of the *Picornaviridae* family. PICV5 and PICV13 identified in this study were marked with red.

In the present study, 14,045 sequence reads matched to the *Hepatovirus* genus were obtained in the library. Through sequence splicing, two complete genomes of hepatoviruses were acquired and temporarily named PICV5 (mean coverage: 2,735.3) and PICV13 (mean coverage: 342.8), respectively. The genome of PICV5 and PICV13 is 7,871 and 7,754 nt in length, with the GC content of 44.5% and 45.4%. Both of them have one ORF encoding 2,241 aa and 2,198 aa of polyprotein, with different lengths of UTRs at both ends of their genomes. Similar to other hepatoviruses, the polyproteins of PICV5 and PICV13 can be cleaved into VP4, VP2, VP3, VP1, 2A–2C, and 3A–3D via a comparison with the polyprotein of the Tupaia hepatovirus A isolate TN1 (NC_028981) (Figure 3A). The P1 polypeptides of PICV5 and PICV13 are composed of 800 aa and 802 aa and undergo cleavage at the VP4/VP2 (Q^38^/D^39^ and Q^36^/D^37^), VP2/VP3 (M^260^/G^261^ and M^260^/M^261^), and VP3/VP1 (T^512^/S^513^ and G^511^/N^512^). The P2 polypeptides of PICV5 and PICV13 consist of 660 aa and 637 aa and contain three nonstructural proteins, including 2A (cleavage sites: E^1,020^/T^1,021^ and D^999^/V^1,000^), 2B (cleavage sites: Q^1,126^/S^1,127^ and Q^1,106^/S^1,107^), and 2C. In the 2C protein of PICV5 and PICV13, there is a conserved NTPase motif (Figure 3A). The P3 polypeptides of PICV5 and PICV13 are 781 aa and 759 aa in length and cleaved into four nonstructural proteins including 3A, 3B, 3C*^pro^*, and 3D*^pol^* at sites 3A/3B (K^1,518^/K^1,519^ and T^1,505^/M^1,506^), 3B/3C (Q^1,544^/S^1,545^ and Q^1,520^/S^1,521^), and 3C/3D (N^1,578^/S^1,579^ and N^1,735^/S^1,736^) (Supplementary Table 1). Some conserved proteinase and polymerase motifs, including GXCGX_10–15_GXH, KDE, PSG, and FLKR, were presented in the 3C and 3D proteins (Figure 3A; Supplementary Table 2).

**FIGURE 4:**
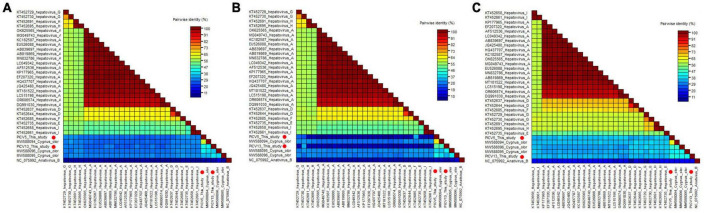
Pairwise comparison of amino acid sequences of two hepatoviruses identified in this study with the representative strains of different species of the genus *Hepatovirus.* **(A)** Pairwise comparison based on polyproteins. **(B)** Pairwise comparison based on P1. **(c)** Pairwise comparison based on the regions of 2C+3CD. PICV5 and PICV13 identified in this study were marked with red solid circle.

Phylogenetic analysis was performed based on the P1 region and 3CD of PICV5, PICV13, and other representative strains belonging to the *Hepatovirus* genus (Figures 3B, C). The results showed that PICV5 and PICV13 clustered with other novel hepatoviruses (MW588094, MW588095, and MW588096) isolated from fecal samples of *Cygnus olor* in the United Kingdom in 2016, forming a separate branch that was far away from other branches formed by known hepatovirus species. To determine if they represented a new species, amino acid sequence alignment was performed among polyproteins, P1, and “2C+3CD” of PICV5 and PICV13 with the same regions of other representative strains belonging to different species of the *Hepatovirus* genus (Figure 4). The polyprotein alignment result showed that PICV5 and PICV13 shared the highest aa identity of 60.3% and 41.1% with the strain MW588094, respectively, while < 30% with other representative strains belonging to the *Hepatovirus* genus (Figure 4A). The P1 alignment indicated that PICV5 and PICV13 have the highest aa identities of 65.5% and 46.9% with the strain MW588094, respectively, while < 33% with other representative strains (Figure 4B). The alignment result based on the “2C+3CD” region showed that PICV5 and PICV13 shared the highest aa identity of 63.4% and 43.4% with the strain MW588094, respectively, while < 36.5% with other representative strains (Figure 4C). Based on the ICTV classification standard, PICV5 and PICV13 should be classified as novel species of the *Hepatovirus* genus.

### Four novel megriviruses coevolve in bar-headed goose

**FIGURE 5:**
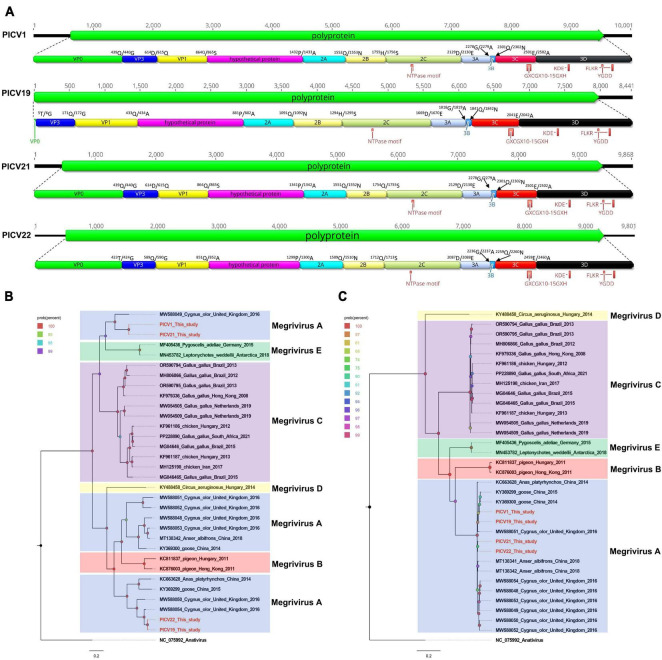
The genomic organization, conserved motifs, and phylogenetic analysis of these megriviruses identified in bar-headed geese. **(A)** The genomic organization of four megriviruses strains. The ORFs and viral encoding proteins of megriviruses were marked with different colors. The conserved motifs were also shown. **(B,C)** The phylogenetic analysis based on the P1 region and 3CD of megriviruses, which identified in this study, and other reference strains belonging to the *Megrivirus* genus of the *Picornaviridae* family. PICV1, PICV19, PICV21 and PICV22 identified in this study were marked with red.

In this study, 25,690 sequence reads belonging to the *Megrivirus* genus were identified. Through sequence splicing, four nearly complete genomes were obtained and temporarily named PICV1 (mean coverage: 735.8), PICV19 (mean coverage: 529.3), PICV21 (mean coverage: 661.7), and PICV22 (mean coverage: 403.4). The genomes of megrivirus PICV1, PICV19, PICV21, and PICV22 are 10,001 nt, 8,441 nt, 9,868 nt, and 9,801 nt in length, respectively, each containing a single ORF encoding one polyprotein (Figure 5A). Like other megriviruses, the polyproteins of PICV1, PICV19, PICV21, and PICV22 are cleaved into VP0, VP3, VP1, hypothetical protein, 2A–2C, and 3A–3D via a comparison with the polyprotein of the chicken picornavirus 4 isolate 5C (NC_024768) (Figure 5A). The P1 polypeptides of PICV1, PICV21, and PICV22 are 1,341 aa, 1,341 aa, and 1,299 aa in length. The P1 polypeptide of partial fragments missing PICV1 is 881 aa in length. Both their P1 polypeptides undergo cleavage at the VP0/VP3 (Q^439^/G^440^, T^5^/G^6^, Q^439^ /G^440^, and T^423^/G^424^), VP3/VP1 (D^614^/Q^615^, Q^171^/G^172^, Q^614^/G^615^, and T^589^/G^590^), and VP1/hypothetical protein (Q^864^/S^865^, Q^433^/A^434^, Q^864^/S^865^, and Q^851^/A^852^). All P2 polypeptides of these megriviruses are 788 aa and consist of three nonstructural proteins, including 2A (cleavage site: Q^1,552^/N^1,553^, Q^1,091^/N^1,092^, Q^1,552^/N^1,553^, and Q^1,509^/N^1,510^), 2B (cleavage site: H^1,755^/S^1,756^, H^1,294^/S^1,295^, H^1,755^/S^1,756^, and Q^1,712^/S^1,713^), and 2C. A conserved NTPase motif is presented in their 2C protein (Figure 5A). The P3 polypeptides of PICV1, PICV19, PICV21, and PICV22 are 846 aa in length and cleaved into four nonstructural proteins including 3A, 3B, 3C*^pro^*, and 3D*^pol^* at sites 3A/3B (G^2278^/A^2279^, G^1818^/A^1819^, G^2,278^/A^2,279^, and G^2,236^/A^2,237^), 3B/3C (Q^2,301^/N^2,302^, Q^1,841^/N^1,842^, Q^2,301^/N^2,302^, and Q^2,259^/N^2,260^), and 3C/3D (E^2,501^/A^2,502^, E^2,041^/A^2,042^, E^2,501^/A^2,502^, and E^2,459^/A^2,460^) (Supplementary Table 1). In their 3C and 3D proteins, there are some conserved proteinase and polymerase motifs, including GXCGX_10–15_GXH, KDE, FLKR, and FLKR (Figure 5A; Supplementary Table 2).

**FIGURE 6:**
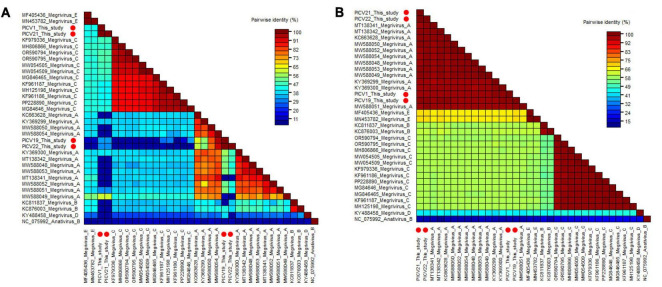
Pairwise comparison of amino acid sequences of four megriviruses identified in this study with the representative strains of different species of the genus *Megrivirus.* **(A)** Pairwise comparison based on P1. **(B)** Pairwise comparison based on the regions of 2C+3CD. PICV1, PICV19, PICV21 and PICV22 identified in this study were marked with red solid circle.

Two phylogenetic trees were constructed based on the P1 region and 3CD of PICV1, PICV19, PICV21, and PICV22, and the same regions of other representative megriviruses (Figures 5B, C). The result of the P1 phylogenetic tree showed that PICV1 and PICV21 clustered with the Megrivirus A strain MW588049 isolated from fecal samples of *Cygnus olor* in the United Kingdom in 2016, forming a separate branch, while PICV19 and PICV21 clustered with other Megrivirus A strains (MW588050, MW588054, KY369299, and KC663628) identified from fecal samples of *Cygnus olor*, goose, and duck, forming another branch. In the 3CD phylogenetic tree, PICV1, PICV19, PICV21, and PICV22 clustered with other Megrivirus A strains, forming a branch. According to the above results, we think that the 3CD region of megriviruses is more suitable for studying virus evolution. To determine whether these four viruses belong to the Megrivirus A species, amino acid sequence alignment was performed among P1 and “2C+3CD” of PICV1, PICV19, PICV21, and PICV22 with the same regions of other representative strains belonging to different species of the *Megrivirus* genus (Figure 6). The P1 alignment result showed that PICV1 and PICV21 shared the highest aa identity of 59.6% with the strain MW588049, while PICV19 and PICV22 had the highest aa identity of 95.6% and 93.1% with the strain MW588054. The alignment result based on the “2C+3CD” region showed that PICV1, PICV19, PICV21, and PICV22 shared aa identity of > 97% with other representative strains of the Megrivirus A species. Based on the ICTV classification standard, PICV1, PICV19, PICV21, and PICV22 should be classified as novel strains of the Megrivirus A species.

**FIGURE 7:**
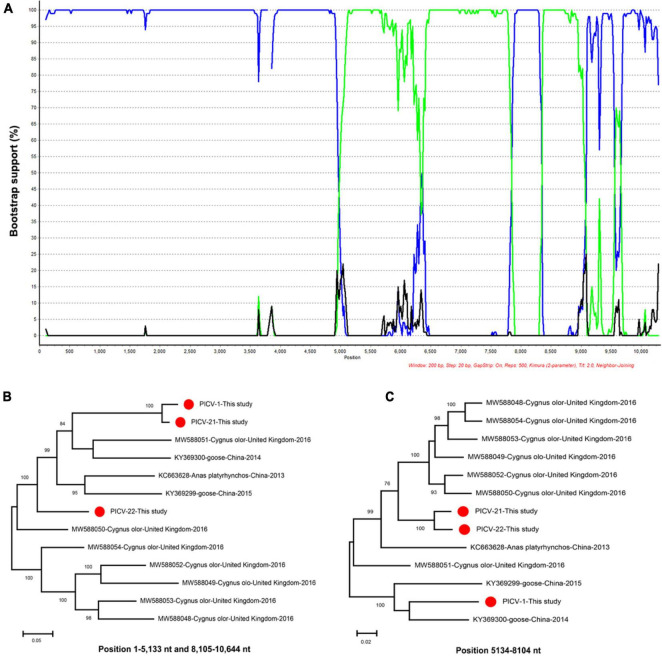
Recombination and phylogenetic analysis of megriviruses from bar-headed geese. **(A)** BOOTSCAN evidence for the recombination origin on the basis of pairwise distance, modeled with a window size 200, step size 20, and 100 Bootstrap replicates. **(B)** Neighbor-joining tree constructed using the region of 1–5,133 nt and 8,105–10,644 nt. **(C)** Neighbor-joining tree constructed using the region of 5,134–8,104 nt. Neighbor-joining method based on 1,000 replicates using MEGA-X software.

Recombinant analysis was performed based on the genome sequences of PICV1, PICV21, and PICV22, along with those of other Megrivirus A strains Using the RDP5, The result of the recombination analysis showed that the PICV21 strain was a recombinant, and the major and minor parent were PICV1 and PICV22, respectively (Supplementary Figure 1). We further confirmed the recombinant result using the BootScan program in the SimPlot software v3.5.1 (Figure 7A). The recombination event was also confirmed by phylogenetic analysis. Two phylogenetic trees were constructed based on the genome regions of 1–5,133 nt and 8,105–10,644 nt, and 5,134–8,104 nt, respectively. The tree based on 1–5,133 nt and 8,105–10,644 nt showed that PICV21 clustered with the PICV1, while the tree based on 5,134–8,104 nt showed that PICV21 clustered with the PICV22 (Figures 7B, C).

## Discussion

Bar-headed geese, one of the most well-known high-altitude bird species, has become renowned as an example of high-altitude adaptation. As a migratory bird, it may spread viruses during migration, leading to diseases in birds and humans (Wang et al., 2023). Although previous studies showed that the highly pathogenic avian influenza virus could infect bar-headed geese and cause their deaths (Cao et al., 2019; Meng et al., 2019; Pawar et al., 2023). However, there is limited knowledge about the other viruses that bar-headed geese may carry and infect. These facts highlight the importance of current research in monitoring the potential viral threats of wild bar-headed geese to wildlife and humans.

In this study, we detected and sequenced seven picornaviruses from the sampled bar-headed geese, with most of the viruses being genetically diverse from the other currently known viruses. These picornaviruses were classified into three different genera, including the *Anativirus* genus, the *Hepatovirus* genus, and the *Megrivirus* genus. So many different types of picornaviruses have been detected in the bar-headed geese, indicating that the bar-headed geese are susceptible hosts of picornaviruses.

*Anativirus* is a new genus of the family *Picornaviridae* and is classified into two species, including Anativirus A and Anativirus B, which mainly infect chickens and ducks (Vibin et al., 2020). Members of the Anativirus A species were mainly detected in duck samples, while the Anativirus B strains were mostly identified in chicken samples (Tseng and Tsai, 2007; Boros et al., 2016). In this study, one anativirus was first detected in fecal samples of bar-headed geese and temporarily named PICV4. PICV4 showed a closer relationship with the strain MW588168 isolated from a fecal sample of *Cygnus olor* in the United Kingdom, while being relatively distant from other representative strains of the *Anativirus* genus and the *Sapelovirus* genus based on the results, regardless of sequence alignment or evolutionary analysis. According to the International Committee on Taxonomy of Viruses (ICTV) classification for picornaviruses,^1^ PICV4 and the strain MW588168 can be considered new species of the *Anativirus* genus. Currently, some novel anativiruses have been isolated from different genera of the *Anatidae* family. This prompted us to think that novel anativiruses can infect a broad range of hosts. However, the epidemiological and pathological characteristics of anativiruses are not well understood. Therefore, further experimental and epidemiological studies are needed to understand their transmission mechanisms and host species spectrum.

*Hepatovirus* is a genus of single-stranded RNA viruses in the *Picornaviridae* family that infect vertebrates. In humans, the hepatitis A virus (HAV) causes mild hepatitis (Pintó et al., 2021). In recent years, HAV-like viruses have been detected in multiple animals and birds, including seals, bats, rodents, hedgehogs, shrews, and *Cygnus olors* (Anthony et al., 2015; Drexler et al., 2015; Li et al., 2018). The identification of PICV5 and PICV13 as novel Hepatovirus species associated with bar-headed geese adds to the growing evidence of hepatoviruses in diverse hosts beyond mammals, including birds. Their clustering with other recently discovered strains from *Cygnus olor* suggests a potential host specificity for this swan species and hints at a complex ecological network of virus circulation within waterfowl populations. We cannot rule out the possibility that these viruses may come from contaminated food or water. Additional sampling of blood and tissue from bar-headed geese for virus testing would prove beneficial in ascertaining whether they can be infected by the hepatoviruses PICV5 and PICV13. Given the clinical significance of hepatoviruses such as HAV in human health, understanding the prevalence, host range, and evolutionary relationships of these novel strains is crucial for assessing their zoonotic potential and implementing appropriate preventive measures.

Megrivirus has been detected in fecal and respiratory samples of healthy and diseased poultry and birds in previous studies (Honkavuori et al., 2011; Liao et al., 2014; Boros et al., 2017; Gerber et al., 2019; Yinda et al., 2019; Kwok et al., 2020; Haji Zamani et al., 2023). In the present study, the identification and characterization of four novel megrivirus strains, PICV1, PICV19, PICV21, and PICV22, from avian fecal samples represent a significant contribution to our understanding of the genetic diversity and evolution within the *Megrivirus* genus. These findings underscore the ubiquitous nature of megriviruses in avian populations, with implications for both poultry health and viral ecology. The near-complete genomic sequencing and subsequent phylogenetic analysis reveal that these strains are closely related to known Megrivirus A species, especially those isolated from *Cygnus olor* and other waterfowl species. The high amino acid identity observed in the “2C+3CD” region among the studied strains and other Megrivirus A representatives supports their classification as novel strains within the Megrivirus A species, adhering to ICTV guidelines. This consistency in the nonstructural region suggests that the 3CD portion might be a more reliable marker for studying the evolutionary relationships among megriviruses. However, the notable discrepancy in the P1 region, with PICV1 and PICV21 clustering separately from PICV19 and PICV22, introduces complexity to the evolutionary narrative. This variation could indicate differential selective pressures acting on the structural proteins, potentially linked to host adaptation or virus-host interactions. The detection of a recombination event in PICV21, with PICV1 and PICV22 serving as the major and minor parents, respectively, provides a fascinating insight into the mechanisms driving viral diversification within the genus. Recombination is a key driver of genetic innovation in RNA viruses and can lead to the emergence of novel strains with altered host ranges, pathogenicity, or transmissibility. This finding highlights the dynamic nature of megrivirus evolution and underscores the importance of comprehensive genomic surveillance to monitor potential changes that could impact poultry health or pose zoonotic threats.

The study investigating the diversity of picornaviruses in bar-headed geese has indeed contributed significantly to our knowledge base regarding avian virology and the potential impact of these viruses on wildlife health. However, to achieve a comprehensive understanding and ensure the reliability of the findings, it is imperative to critically assess the limitations of this research. Firstly, the study utilized a relatively small number of fecal samples collected from bar-headed geese. This sample size, while sufficient for preliminary insights, may not accurately reflect the true prevalence or genetic diversity of picornaviruses within the entire population. Larger sample sizes, ideally covering a broader range of age groups, sexes, and health statuses, would provide more robust data. Secondly, the geographical scope of the sample collection might not adequately encompass the full migratory range of bar-headed geese. These birds are known for their extensive migrations across Asia, and different regions might harbor distinct viral strains due to environmental and ecological variations. To mitigate this issue, future studies should strive for a more comprehensive sampling strategy that includes multiple locations along the birds’ migratory paths. This approach would help in understanding the spatial dynamics of picornavirus infections and their potential spread between different ecosystems.

In summary, this study demonstrates that bar-headed geese harbor a variety of genetically diverse picornaviruses, some of which may represent novel species, highlighting the need for further investigation into their pathogenic, epidemiological, and ecological characteristics. Such research is vital for evaluating the potential impacts on poultry and humans and devising appropriate control strategies. Future investigations should prioritize understanding the viruses’ pathogenicity, transmission routes, and natural ecological cycles, particularly within bar-headed goose populations and their potential spillover into domestic animals and humans.

## Data availability statement

The datasets presented in this study can be found in online repositories. The names of the repository/repositories and accession number(s) can be found below: https://www.ncbi.nlm.nih.gov/genbank/, PP827029 to PP827035, https://www.ncbi.nlm.nih.gov/genbank/, SRR29463689.

## Ethics statement

The study was approved by the Jiangsu University Ethics Committee on the use of animals and complied with Chinese ethics laws and regulations.

## Author contributions

YS: Conceptualization, Writing−original draft. YW: Investigation, Methodology, Validation, Writing−review and editing. LJ: Investigation, Methodology, Validation, Writing−review and editing. QZ: Investigation, Methodology, Validation, Writing−review and editing. QS: Investigation, Methodology, Validation, Writing−review and editing. XW: Investigation, Methodology, Validation, Writing−review and editing. YL: Investigation, Methodology, Validation, Writing−review and editing. LkJ: Investigation, Methodology, Validation, Writing−review and editing. SY: Supervision, Conceptualization, Data curation, Formal analysis, Funding acquisition, Investigation, Project administration, Resources, Validation, Writing−original draft, Writing−review and editing. WZ: Conceptualization, Data curation, Formal analysis, Funding acquisition, Investigation, Project administration, Resources, Supervision, Validation, Writing−review and editing.

## Footnotes

1
https://ictv.global/report/chapter/picornaviridae/anativirus

## Supplementary material

Supplementary Figure 1 The recombination information and the RDP evidence for recombination event. **(a)** The detailed recombination information was shown including the recombinant, minor and major parents, beginning and ending breakpoints, and the P-val of eight testing methods. **(b)** RDP evidence for the recombination origin on the basis of pairwise identity.
