# Supplementary material for: Identification and characterization of multiple novel picornaviruses in fecal samples of bar-headed goose
Source: Front Microbiol. 2024 Jul 26;15:1440801. doi: 10.3389/fmicb.2024.1440801 (PMC11310119; doi:10.3389/fmicb.2024.1440801)
Supplement: Supplementary file 1 [file Table_1.DOCX]

Table. S1. The cleavage sites of polyproteins of different picornaviruses in this study.

| Protein | PICV-1 | |  | PICV-19 | |  | PICV-21 | |  | PICV-22 | |
| --- | --- | --- | --- | --- | --- | --- | --- | --- | --- | --- | --- |
|  | Position | cleavage sites |  | Position | cleavage sites |  | Position | cleavage sites |  | Position | cleavage sites |
| VP3 | 440~614 | 439/440 (Q/G) |  | 6~171 | 5/6  (T/G) |  | 440~614 | 439/440 (Q/G) |  | 424~589 | 423/424 (Q/G) |
| VP1 | 615~864 | 614/615 (D/Q) |  | 172~433 | 171/172 (Q/G) |  | 615~864 | 614/615 (D/Q) |  | 590~851 | 589/590 (Q/G) |
| Hypothetical protein | 865~1432 | 864/865 (Q/S) |  | 434~881 | 433/434 (Q/A) |  | 865~1341 | 864/865 (Q/S) |  | 852~1299 | 851/852 (D/Q) |
| 2A | 1433~1552 | 1432/1433 (P/A) |  | 882~1091 | 881/882 (P/A) |  | 1342~1551 | 1341/1342 (P/A) |  | 1300~1509 | 1299/1300 (Q/S) |
| 2B | 1553~1755 | 1552/1553 (Q/S) |  | 1092~1294 | 1091/1092 (Q/N) |  | 1552~1754 | 1551/1552 (Q/N) |  | 1510~1712 | 1509/1510 (P/A) |
| 2C | 1756~2129 | 1755/1756  (H/S) |  | 1295~1669 | 1294/1295  (H/S) |  | 1755~2129 | 1754/1755  (Q/S) |  | 1713~2087 | 1712/1713 (Q/N) |
| 3A | 3130~2278 | 2129/2130  (D/E) |  | 1670~1818 | 1669/1570  (D/E) |  | 2130~2278 | 2129/2130  (D/E) |  | 2088~2236 | 2087/2088  (Q/S) |
| 3B | 2279~2301 | 2278/2279 (G/A) |  | 1819~1841 | 1818/1819 (G/A) |  | 2279~2301 | 2278/2279 (G/A) |  | 2237~2259 | 2236/2237  (D/E) |
| 3C | 2302~2501 | 2301/2302 (Q/N) |  | 1842~2041 | 1841/1842 (Q/N) |  | 2302~2501 | 2301/2302 (Q/N) |  | 2260~2459 | 2259/2260 (G/A) |
| 3D | 2502~2975 | 2501/2502 (E/A) |  | 2042~2515 | 2041/2042 (E/A) |  | 2502~2975 | 2501/2502 (E/A) |  | 2460~2933 | 2459/2460 (Q/N) |
| Protein | PICV-4 | |  | PICV-5 | |  | PICV-13 | |  |  | |
|  | Position | cleavage sites |  | Position | cleavage sites |  | Position | cleavage sites |  |  |  |
| VP4 | 448~512 | 447/448  (Q/S) |  | 1~38 | - |  | 1~36 | - |  |  |  |
| VP0 | - | - |  | 39~260 | 38/39  (Q/D) |  | 37~260 | 36/37  (Q/D) |  |  |  |
| VP2 | 513~766 | 512/513  (Q/N) |  | - | - |  | - | - |  |  |  |
| VP3 | 767~1009 | 766/767  (Q/G) |  | 261~512 | 260/261 (M/G) |  | 261~511 | 260/261  (M/M) |  |  |  |
| VP1 | 1010~1289 | 1009/1010  (E/G) |  | 513~800 | 512/513 (T/S) |  | 512~802 | 511/512  (G/N) |  |  |  |
| 2A | 1290~1297 | 1289/1290  (I/A) |  | 801~1020 | 800/801 (D/N) |  | 803~999 | 802/803  (E/S) |  |  |  |
| 2B | 1298~1405 | 1297/1298  (Q/G) |  | 1021~1126 | 1020/1021 (E/T) |  | 1000~1106 | 999/1000  (Q/V) |  |  |  |
| 2C | 1406~1738 | 1405/1406  (Q/G) |  | 1127~1460 | 1126/1127 (Q/S) |  | 1107~1439 | 1106/1107  (Q/S) |  |  |  |
| 3A | 1739~1843 | 1738/1739  (Q/G) |  | 1461~1517 | 1460/1461  (Q/S) |  | 1440~1505 | 1439/1440  (Q/A) |  |  |  |
| 3B | 1844~1865 | 1843/1844  (Q/G) |  | 1518~1544 | 1517/1518  (K/K) |  | 1506~1520 | 1505/1506  (T/M) |  |  |  |
| 3C | 1866~2050 | 1865/1866  (Q/G) |  | 1545~1758 | 1544/1545 (Q/S) |  | 1521~1735 | 1520/1521  (Q/S) |  |  |  |
| 3D | 2051~2515 | 2050/2051  (E/G) |  | 1759~2241 | 1758/1759 (N/S) |  | 1736~2198 | 1735/1736  (N/S) |  |  |  |
