# Supplementary material for: Identification and characterization of multiple novel picornaviruses in fecal samples of bar-headed goose
Source: Front Microbiol. 2024 Jul 26;15:1440801. doi: 10.3389/fmicb.2024.1440801 (PMC11310119; doi:10.3389/fmicb.2024.1440801)
Supplement: Supplementary file 2 [file Table_2.DOCX]

Table. S2. The conservative motifs of different picornaviruses in this study

| Motif | PICV-1 | |  | PICV-19 | |  | PICV-21 | |  | PICV-22 | |
| --- | --- | --- | --- | --- | --- | --- | --- | --- | --- | --- | --- |
|  | Position | AA sequence |  | Position | AA sequence |  | Position | AA sequence |  | Position | AA sequence |
| NTPase motif | 1882~1889 | GGPGCGKS |  | 1422~1429 | GGPGCGKS |  | 1882~1889 | GGPGCGKS |  | 1840~1847 | GGPGCGKS |
| GXCGX10-15GXH | 2458~2478 | GMCGSPLLSTNSAREVVLGIH |  | 1998~2018 | GMCGSPLLSTNSAREVVLGIH |  | 2458~2478 | GMCGSPLLSTNSAREVVLGIH |  | 2416~2436 | GMCGSPLLSTNSAREVVLGIH |
| KDE | 2667~2669 | KDE |  | 2207~2210 | KDE |  | 2667~2670 | KDE |  | 2625~2627 | KDE |
| YGDD | 2836~2839 | YGDD |  | 2376~2379 | YGDD |  | 2836~2839 | YGDD |  | 2794~2797 | YGDD |
| FLKR | 2885~2888 | FLKR |  | 2425~2428 | FLKR |  | 2885~2888 | FLKR |  | 2843~2845 | FLKR |
| Motif | PICV-4 | |  | PICV-5 | |  | PICV-13 | |  |  | |
|  | Position | AA sequence |  | Position | AA sequence |  | Position | AA sequence |  |  |  |
| NTPase motif | 1537~1544 | GKPGCGKS |  | 1265~1272 | GPRGCGKS |  | 1244~1251 | GPRGSGKS |  |  |  |
| GXCGX10-15GXH | 2012~2028 | GQCGGLICKAGQVLGIH |  | 1710~1731 | GSCGGVLISSNNRLGNPFIGIH |  | 1686~1707 | GSCGGILISANNKIGCPFVGAH |  |  |  |
| KDE | 2209~2211 | KDE |  | 1923~1925 | KDE |  | 1900~1902 | KDE |  |  |  |
| PSG | 2340~2342 | PSG |  | 2057~2059 | PSG |  | 2034~2036 | PSG |  |  |  |
| YGDD | 2340~2342 | YGDD |  | 2099~2102 | YGDD |  | 2076~2079 | YGDD |  |  |  |
| FLKR | 2379~2382 | FLKR |  | 2150~2153 | FLKR |  | 2127~2130 | FLKR |  |  |  |
