# Supplementary figures and images for: Identification and characterization of multiple novel picornaviruses in fecal samples of bar-headed goose
Source: Front Microbiol. 2024 Jul 26;15:1440801. doi: 10.3389/fmicb.2024.1440801 (PMC11310119; doi:10.3389/fmicb.2024.1440801)

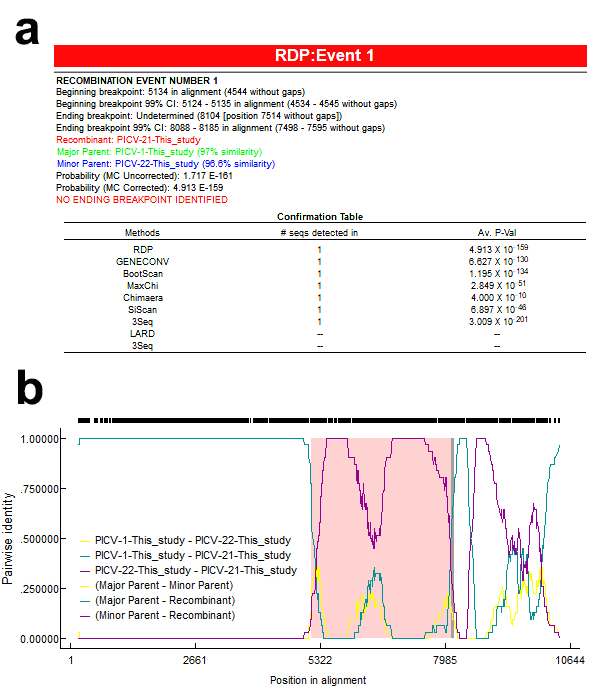

Supplement: Supplementary Figure 1 — The recombination information and the RDP evidence for recombination event. (a) The detailed recombination information was shown including the recombinant, minor and major parents, beginning and ending breakpoints, and the P-val of eight testing methods. (b) RDP evidence for the recombination origin on the basis of pairwise identity. [file Image_1.JPEG]
